# Improvement of design of a surgical interface using an eye tracking device

**DOI:** 10.1186/1742-4682-11-S1-S4

**Published:** 2014-05-07

**Authors:** Duygun Erol Barkana, Alper Açık

**Affiliations:** 1 Electrical and Electronics Engineering Department, Yeditepe University, Istanbul, 34755, Turkey; 2 Psychology Department, Özyeğin University, Istanbul, 34794, Turkey

**Keywords:** surgical interface, human factor analysis, user-centered approach, eye tracking, mental workload analysis, situation awareness

## Abstract

**Background:**

Surgical interfaces are used for helping surgeons in interpretation and quantification of the patient information, and for the presentation of an integrated workflow where all available data are combined to enable optimal treatments. Human factors research provides a systematic approach to design user interfaces with safety, accuracy, satisfaction and comfort. One of the human factors research called user-centered design approach is used to develop a surgical interface for kidney tumor cryoablation. An eye tracking device is used to obtain the best configuration of the developed surgical interface.

**Methods:**

Surgical interface for kidney tumor cryoablation has been developed considering the four phases of user-centered design approach, which are analysis, design, implementation and deployment. Possible configurations of the surgical interface, which comprise various combinations of menu-based command controls, visual display of multi-modal medical images, 2D and 3D models of the surgical environment, graphical or tabulated information, visual alerts, etc., has been developed. Experiments of a simulated cryoablation of a tumor task have been performed with surgeons to evaluate the proposed surgical interface. Fixation durations and number of fixations at informative regions of the surgical interface have been analyzed, and these data are used to modify the surgical interface.

**Results:**

Eye movement data has shown that participants concentrated their attention on informative regions more when the number of displayed Computer Tomography (CT) images has been reduced. Additionally, the time required to complete the kidney tumor cryoablation task by the participants had been decreased with the reduced number of CT images. Furthermore, the fixation durations obtained after the revision of the surgical interface are very close to what is observed in visual search and natural scene perception studies suggesting more efficient and comfortable interaction with the surgical interface. The National Aeronautics and Space Administration Task Load Index (NASA-TLX) and Short Post-Assessment Situational Awareness (SPASA) questionnaire results have shown that overall mental workload of surgeons related with surgical interface has been low as it has been aimed, and overall situational awareness scores of surgeons have been considerably high.

**Conclusions:**

This preliminary study highlights the improvement of a developed surgical interface using eye tracking technology to obtain the best SI configuration. The results presented here reveal that visual surgical interface design prepared according to eye movement characteristics may lead to improved usability.

## Background

Increasing the speed of the surgical process and decreasing the level of invasiveness are two important objectives in a surgical procedure along with a successful treatment of the diseased lesion. Minimally invasive surgical procedures have been evolved for reducing hospitalization time and surgical complexities. In a conventional minimally invasive surgery however, a surgeon operates on deeply located lesions without actually seeing or touching. Thus, an easy to use surgical interface (SI), which benefits maximally from the surgeons' skills while providing all necessary information that can be perceived and processed by the surgeon during the intervention in the operation theatre, is needed. Surgical interfaces are designed to improve surgical treatments in all the stages of a clinical workflow, which ranges from preoperative diagnosis and planning of the surgical interventions up to postoperative evaluation. In this work, SI is used for the interpretation and quantification of the patient information, and for the presentation of an integrated workflow where all available data are combined to enable optimal treatments. Recently, several SIs that consist of functions for identification of liver segments and planning of liver surgery have been developed.

CAScination is a well-known commercial system that integrates stereotactic technology in complex liver interventions and surgery [1]. The real-time visualization of surgical tools of the surgical interface shows the surgeon where to cut/ablate [2]. A preoperative surgical simulator has been designed to allow surgeons to plan the surgical interventions for liver surgery in PAtient Specific Simulation and PreOperative Realistic Training (PASSPORT) project [2]. A dynamic liver modeling can be developed with the help of a preoperative surgical planning simulator in PASSPORT. Liver pre-operative images, surrounding anatomical and pathological structures are used to extract anatomical (from CT-image, MRI or US), mechanical (from elastographic imaging) and biological information of liver (from biopsy and blood analysis). LiverAnalyzer™ (MeVis Medical Solutions AG, Germany) and Synapse Vincent™ (FUJIFILM Co., Japan) surgical interfaces have functions that can segment the liver, vessels, biliary system, and tumors, volumetry of the remnant and/or graft, evaluate vascular territories, and plan the surgery. Additionally, Pathfinder™ (Pathfinder Technologies, USA) provide a computer-assisted navigation and 3D visualization for surgeons and interventional radiologists, to enable accurate and efficient delivery of cancer therapeutics in soft-tissue organs. E-simulation and planning from radiological exams to surgery (E-SPress3D, Italy) has also been developed to plan and simulate surgical interventions deploying the information containing CT, MRI, 3D-US images. HEPAPLAN (LabHuman Human Centered Technology, Spain) is a software developed to help surgeons for make a better decision about oncological patients in hepatic field. Note that not only inclusion of functions (such as navigation, 3D view etc) for surgery, but also incorporating these functions systematically into interface design by considering the surgeon's requirements is an essential issue. Thus, recently, a user-centered virtual liver surgery planning system for liver surgery called Dr. Liver, has been developed, that considers human factors research, usability and time efficiency issues [3]. Human factors research describes how much and what kind of information a person can use effectively, and how information should be organized and presented to make it usable [4]. The general objectives of the human factors are to maximize user and system efficiency, to increase safety, to improve system performance and to increase ease of use etc. [5].

Human factors research has previously been used to provide design solutions for the disciplines of medicine, psychology and ergonomics in which human-machine interactions affect performance and usability [5]. Various traditional, sociotechnological systems, user-centered design, computer-supported design and ecological interface design approaches in human factors research have been developed to design interfaces [5]. In this work, we use user-centered design (UCD) approach to develop the SI for kidney tumor cryoablation [6],[7]. Foundation principles of UCD is to initially focus on users and tasks, perform empirical measurements by getting user feedback and reactions on design and prototypes, and apply iterative design [8],[9]. One way to improve the design of a visual SI, such as the one developed here, is to investigate the eye movements of users interacting with the interface.

Even though the employment of eye-tracking data in usability research dates back to 1950s [10], due to the slow development of reliable eye tracking technology, and difficulties arising from multidimensional gaze data analysis. Recently there has been an increase in the number of eye tracking studies [11]. Together with research on the experience of pilots training in a simulator [12], users inspecting webpages [13], there have been a couple of studies profiting from eye movement data in medical contexts [14-17]. Nevertheless, those few studies have focused on the visual search strategies of surgeons looking for medically interesting spots in scans [14],[15], or have investigated similarities and differences between novices and experts performing a simulated surgical intervention [17]. What is relevant for our design purposes is the possibility of tracking people's eye movements in order to modify and improve the interface in development. It has long been accepted that aspects of user gaze data are informative in terms of revealing the difficulty of task at hand [11]. One can address the factors that influence the usability of interfaces by deducing users' visual and display-based information processing from eye movement patterns [18]. To our knowledge, no previous study on surgical interface design benefited from eye tracking to improve the arrangement of interface elements displayed on the screen of the surgical interface.

The difficulty that arises while working with eye movements is the multidimensional nature of the data that can be analyzed, and interpreted in several different ways [11]. Eye trackers typically sample the position of the eye(s) in Cartesian coordinates at successive time points. One can usually extract fixation and saccade intervals and obtain several metrics such as fixation durations, amount of fixations in a given region, and the amplitude of saccades using eye tracker data [11], [18]. Thus, there is a need to consider an important trade-off while analyzing eye tracking data obtained from users interacting with a purpose-designed system. While there is a vast amount of fixation and eye-movement related measures one can extract from raw eye position data, comparability across studies requires agreement on few important measures. Nevertheless, unlike free-viewing of natural images [19], where each scene is one representation of the same underlying world, most user interfaces are very different from each other since they are designed for unique tasks. This aspect requires that researchers define particular measures that are best suited for understanding the experience of the user interacting with one particular interface. For instance, if users fixated some regions of the interface significantly more often than others, while ignoring other elements completely, the design of the interface can be improved accordingly. The fixation duration analysis, on the other hand, reflects both the informative region search efficiency of the user, and the amount of information extracted from the regions that are fixated [18]. Thus, while analyzing the data recorded during interaction with a human-computer interaction, the data analysis must employ common gaze measures in a creative and innovative way that serves the purposes of the given interface-based task as well as possible.

How can one use eye movement data in conjunction with task completion time and accuracy in order to evaluate whether a set of changes in the visual appearance of an interface led to improved user experience? Employing ecological tasks such as preparing a cup of tea [20] and relying on natural scene presentations on a screen in the laboratory [21], [22] have demonstrated that every second humans perform on average two to three fast eye movements - saccades - each of which is followed by fixation that lasts around 200-300ms [23]. These eye movements related characteristics thus describe the default mode of active vision. Deviations from these default values while dealing with a task might reflect suboptimal performance. Accordingly, the recorded eye movement properties are compared with the default values to improve the design of our developed SI. In this study, we investigate if the modified version of the SI results in faster and more accurate task completion in the presence of more natural gaze patterns, which can qualify as an objective improvement in usability. Thus, surgeons have been asked to conduct experiments of a simulated cryoablation using the SI with an eye tracker device in order to see whether their eye movements remain natural during this demanding task.

Surgeons are asked to find the tumor on the left kidney (target point) that is displayed on SI, and to determine a suitable entry point to start the ablation. The visual information acquisition, quantified with fixation durations, and amount of fixations at different interface elements (such as menu, 3D images etc), which have been obtained using eye tracker device, are used to improve the design of the SI configuration.

In this article, the Materials and Methods section presents the development of SI considering the four phases of the UCD approach, experimental apparatus, procedure, participants, data collection, analysis. The results of the experiments are discussed in Results section. The Discussion and Conclusion presents the potential contributions of this work. The possible directions for future work are given in Future Work.

## Materials and methods

In this section, initially development of SI considering the UCD approach has been given. Then experimental apparatus, procedure, participants, data collection and analysis details are provided.

### Development of SI using User-Centered Design (UCD) approach

**Figure 1:**
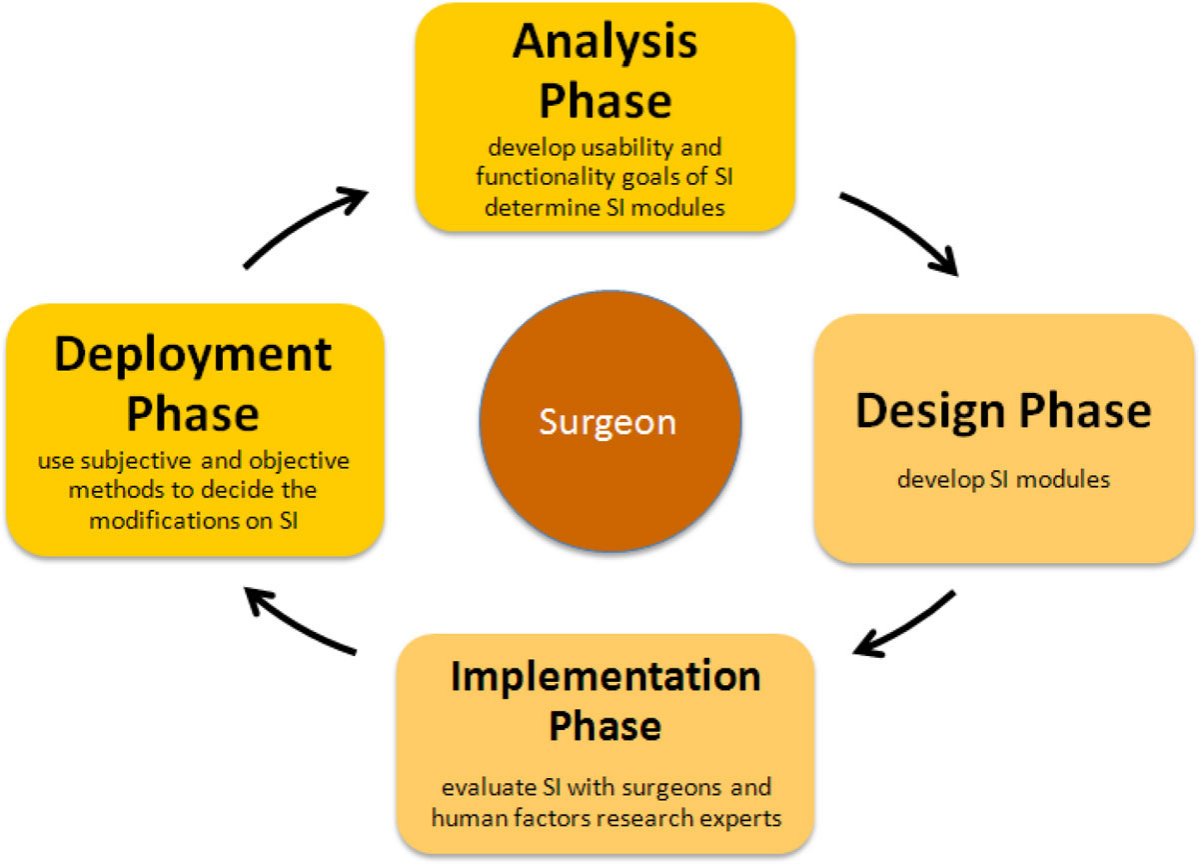
**User-Centered Approach during Design of SI**.

The SI has been designed considering UCD approach (Figure 1). UCD approach consists of four main phases, i) analysis - determine SI modules and define the important usability and functionality factors, ii) design - begin to develop SI prototypes, iii) implementation - construct a heuristic evaluation, whereby usability experts work together with the SI developers and surgeons to analyze the various dimensions of the prototype SIs, and previously-used products with similar functionality of SI, and iv) deployment - use surveys or other evaluation techniques to get surgeons' feedback about SI for modifications. The design details of each phase are given in [6]. The first phase of a UCD approach is the analysis phase. SI modules that satisfy the surgeons' need are determined, and important usability and functionality factors to design these SI modules considering these factors have been defined. 10 surgeons from Turkey have been interviewed to determine what information needs to be included in the SI modules during the operation to satisfy their need. Additionally, total of 12 questionnaires (10 from Turkey, 1 Denmark, 1 Italy) have been collected from the surgeons to investigate the importance of usability and functionality factors for SI from the perspectives of surgeons [24]. Later the SI modules, which are Medical Image, 3D View, Phantom Model, Robot CAD Model, Visualization, Entry-Target Selection, Run-Time (Real-time) and Needle Force Tracking that are defined in the analysis phase have been developed. The details of the modules are given in [6]. The developed SI modules, menu and screen layout are reviewed with the surgeons, and experts in the implementation phase of the UCD approach. A heuristic evaluation, whereby usability experts work together with the SI developers and surgeons to analyze the various dimensions of the prototype SIs, and previously-used products with similar functionality of SI, has been constructed. A usability expert judged whether each element of the SI followed a list of well-established usability heuristics developed by [25],[26]. The heuristic evaluation has been conducted also in the context of typical user tasks to provide feedback to the developers on the extent to which the SI is likely to satisfy the potential surgeons' needs and preferences that have been determined by conducting a survey of selected surgeons. Expert evaluations, which do not use specific heuristics, have also been used. The use of these methods did provide relatively cheap and quick feedback on layout and information structure, consistency of the terminology, use of colors and some other factors to the design team members. The feedback has been provided verbally. The button size, color, font size and information that will be displayed on SI, which form the infrastructure of SI modules, have been decided. The subjective and objective methods, which will be used to modify the SI, are decided in the deployment phase. An eye tracking system, which provides quantitative data, to understand visual and display-based information processing, and the factors, that may impact upon the usability of SI has been used. It is possible to learn where surgeons target on SI using eye tracking system, which will help us to remove the unnecessary information on SI, and obtain the optimum SI configuration.

### Experimental apparatus

**Figure 2:**
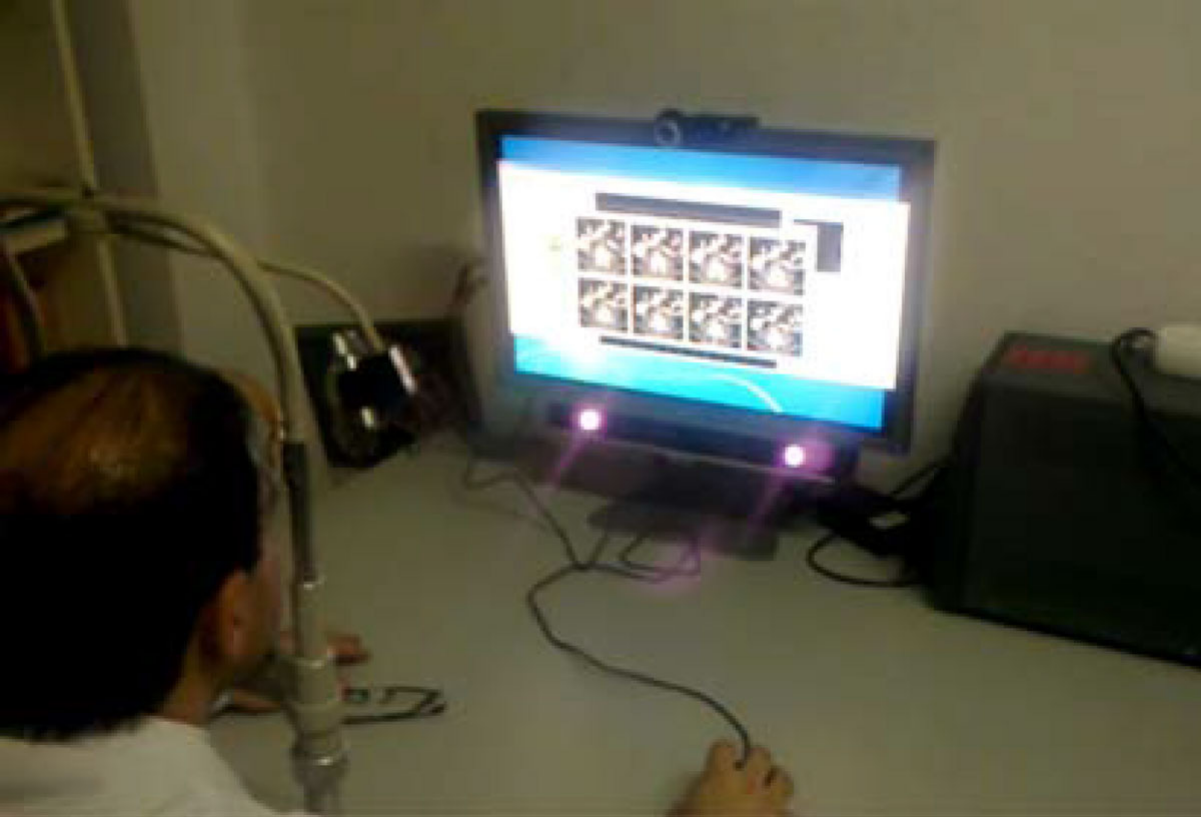
**Surgeon using the early version of the SI**. The SMI eye-tracker is visible below the monitor.

The experimental apparatus shown in Figure 2 consisted of two components. The first component is a personal computer, which created the simulated environment using developed SI to perform the cryoablation of a tumor task. Participants were seated on a non-adjustable chair while viewing the 22" LCD monitor set at 1680 × 1050 resolution of a personal computer from a distance of 70 cm. Thus, the screen covered approximately 37.5° of the visual field horizontally. The second component is a remote eye tracker Sensomotoric Instruments (SMI) 500 for monitoring surgeons' eye motion pattern. The eye tracker control software ran on an HP desktop.

### Participants

Six participants were recruited from Department of Faculty of Medicine, Istanbul University. Given that we are primarily interested in gaze patterns of our participants, this small subject pool was enough to obtain a large set of fixations and eye movements. Three are surgeons (all male) (Participant A (PA), Participant B (PB) and Participant C (PC)) from Urology Department, and three are radiologists (two female and one male) (Participant D (PD), Participant E (PE) and Participant F (PF)) from Radiology Department. All the participants are right-handed, and have normal vision. All urologists have experience with laparoscopic surgery, and radiologists have experience in kidney biopsy process. No participants have any prior experience using an eye tracker device.

### Procedure

Participants were briefed on the nature of the study. The task was to find the tumor on the left kidney (target point), which had been displayed on SI, and to determine a suitable entry point to start the ablation. The participants first took part in a trial practice, during which the participants executed the simple tasks several times to get a basic understanding of the SI. Participants were calibrated and seated with their head resting on the chin-rest to ensure the accuracy of the data recording before the data acquisition was initiated.

**Figure 3:**
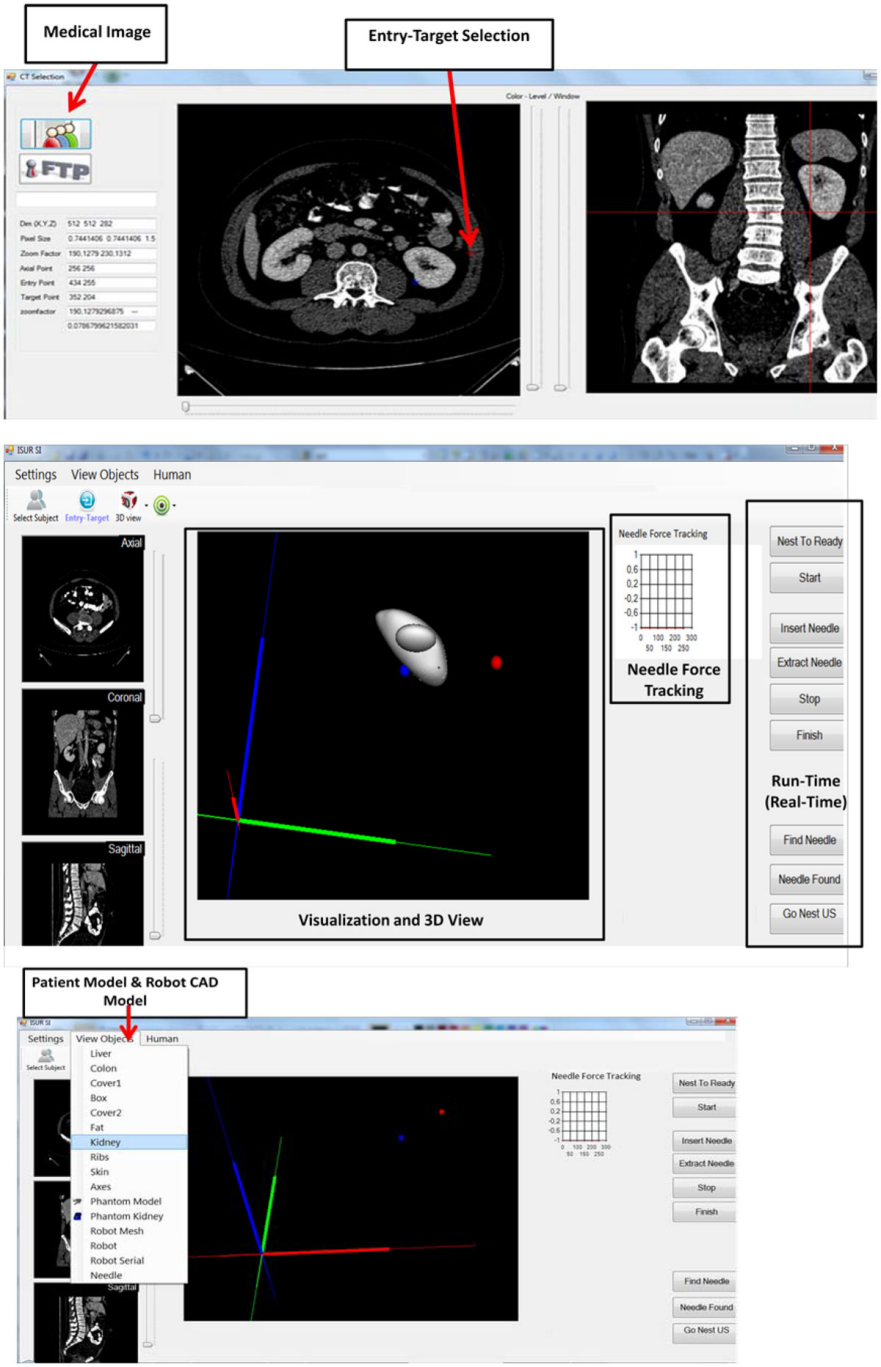
**The modified SI with several modules**.

In the experiment, SI condition was a between-subjects factor with two levels, early SI and the modified SI. In the first SI, eight consecutive CT images taken at successive depth planes were presented simultaneously to the participants (Figure 2), and they were asked to perform the cryoablation task. In the modified SI (Figure 3), only two CT images had been displayed to the participants in the second condition.

Once cryoablation task was completed, the mental workload and situation awareness of the participants have been measured. The National Aeronautics and Space Administration Task Load Index (NASA-TLX) were used to measure the mental workload of the surgeons. Mental workload measures plays an important role in task allocation, and the SI design and development should ensure that the surgeon is not overloaded. NASA-TLX has also been used extensively in a variety of projects for assessing the cognitive load experienced while performing both open and laparoscopic operations [26-29]. The NASA-TLX requires surgeons to rate their perceived levels of mental, physical, and time demands associated with a cryoablation of a tumor task on a scale of 20 points as well as their effort, performance, and frustration during the task execution. Additionally, a subjective technique, namely the Short Post-Assessment Situational Awareness (SPASA), is used for the measurement of situation awareness (SA) [30]. The items contain 11 statements to which the user will express his/her agreement or disagreement on a 4-point Likert scale. Sub-dimensions of SA, which are mission awareness (MA), spatial awareness (SpA), time awareness (TA), perception (L1), comprehension (L2), and prediction (L3), are also captured using these 11 statements.

### Data collection and analysis

The remote eye tracker Sensomotoric Instruments (SMI) 500 sampled the participants' eye position and pupil size at 500 Hz. These data were saved to a log file during the task execution. SMI's control and analysis software Behavioral and Gaze Analysis (SMI BeGaze™, [31]) had been used to extract the gaze related metrics

We had defined task-relevant regions labeled areas of interest (AOIs) on the display and employed three metrics extracted from eye data for the purposes of data analysis. Areas of interest (AOI) on the SI provide a summary of task-related regions including the CT images. In the early version of the SI we defined a total of 11 AOIs, eight of which correspond to the CT scan. The remaining three AOIs were task related buttons, and their data were not analyzed in this study. We defined only two AOIs, since the number of CT scans was reduced to two after analyzing the results obtained with the early SI version in the modified version of the SI. Three eye movement metrics were used to characterize the gaze patterns. Dwell time, the total amount of time spent in an AOI including both fixations and saccades, were used for visualization purposes only. Median fixation durations and AOI specific fixation counts were employed for the statistical analysis. Standard permutation-based bootstrap methods [19] were preferred since they were robust due to the lack of a priori assumptions about the data.

## Results

### Evaluation of early SI results

**Figure 4:**
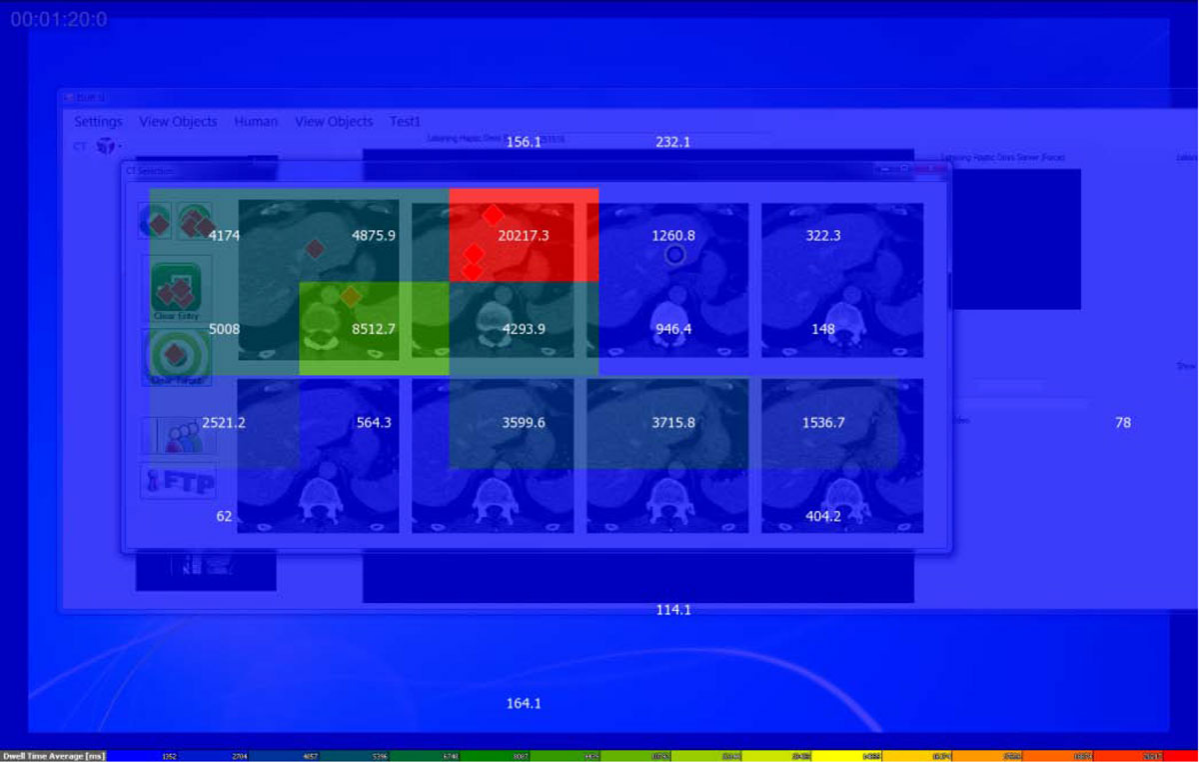
**The early SI version with data obtained from PA**. It can be seen that PA ignored most of the CT scans presented on the SI.

**Figure 5:**
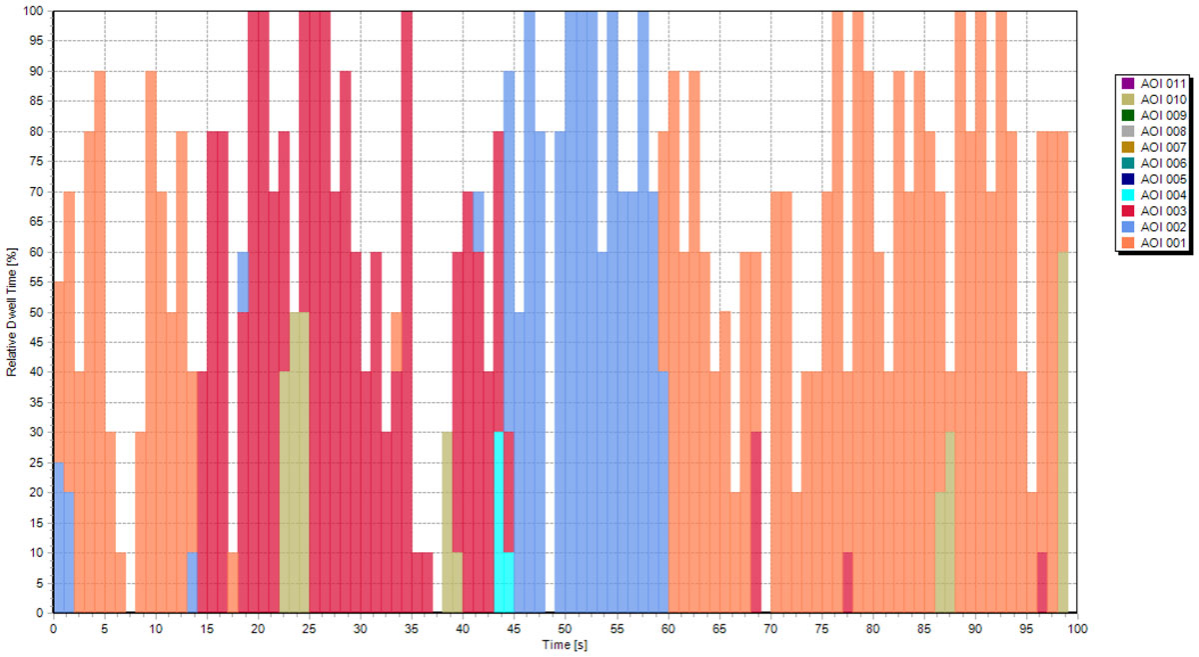
**Temporal evolution of dwell time for PA during interaction with the early SI**.

We investigated if the participants interacting with the early SI version fixated each of the CT scans displayed on the screen, or gather the relevant visual information from few scans only. The data from one representative participant (PA) visualized with SMI BeGaze™ (Figure 4) suggested that most of the CT scans received extremely few fixations. The AOI specific dwell time histograms presented in Figure 4 clearly showed that this participant completed the task while looking at three CT scans. Thus, the visual inspection of the data suggested that most of the CT scans in the early SI version are redundant (Figure 5). It can be seen that PA ignored most of the CT scans presented on the SI.

**Figure 6:**
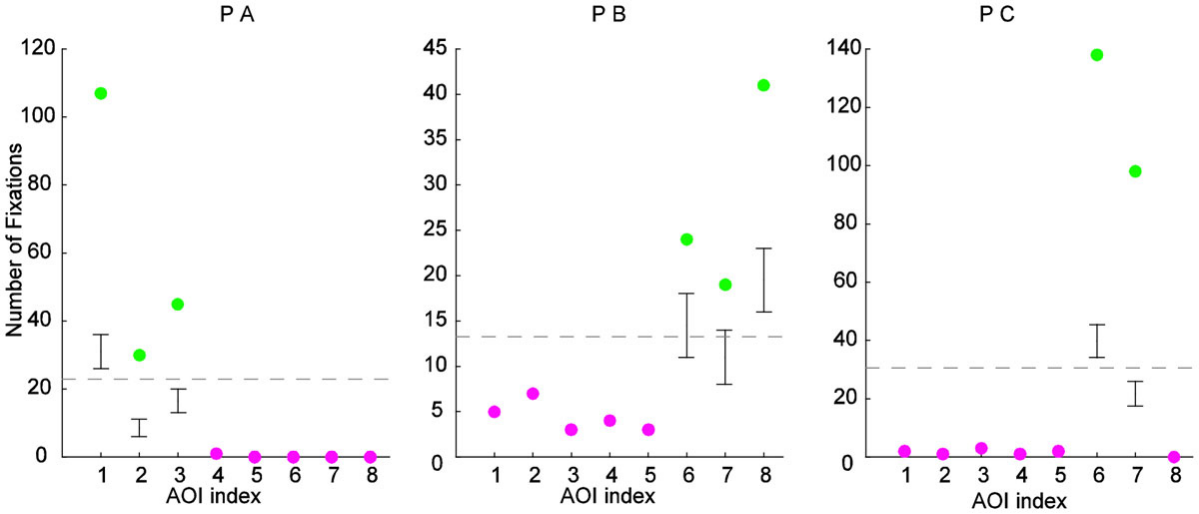
**Participant Specific Fixation Counts during interaction with the early SI**.

The statistical analysis aimed at confirming the intuitions obtained from the visual inspection of the data. First, we had performed a chi-square goodness-of-fit test to see whether the fixation counts in the CT image AOIs were uniformly distributed. This test was performed to provide statistical support for the observation that the participants don't need all of the eight CT images presented on the SI. The three participant-specific tests revealed highly significant deviations from the uniform distribution (PA: χ²(7, N = 189) = 421.50, *p *= 10^-86^; PB: χ²(7, N = 94) = 107.36, *p *= 10^-19^; PC: χ²(7, N = 126) = 512.73, *p *= 10^-105^). The second analysis performed on the data employed bootstrapping techniques in a stairway-like procedure [32]. In order to spot those AOIs that had received more fixations than expected by chance, we had repeated the following steps for each participant: Fixations of a participant were assigned randomly to the eight AOIs, and the maximum fixation count in an AOI reached as a result of this random assignment was recorded. This was performed 10000 times to establish a null-hypothesis distribution for maximum fixation counts. After that, the AOI with the maximum amount of fixations in the original data was tested against the null-distribution. If this maximum fixation count was statistically different than the data gathered in the permutation samples, we had recorded that this AOI was fixated more often than others. In the next step, we had removed the AOI tested in the previous step together with its fixations, and repeated the permutation test for the AOI that had received the second largest amount of fixations. The algorithm stopped when the fixation count in an AOI was not significantly more (*p *> 0.05) than what would be expected by chance. The participant specific fixation counts and the results of the statistical analysis were presented in Figure 6. The circles showed the amount of fixations in each CT image AOI separately for each participant. The gray dashed line showed the uniform distribution. The green dots represented the fixation counts, which are significantly (*p *< 10^-3^) larger than what would be expected by chance. For each significant data-point, the black error bars provided 95% bootstrap confidence intervals (CIs) of the null-hypothesis distribution of maximum fixations in a AOI. Thus, the green dots would be expected to fall into these intervals, if all AOIs had received similar amount of fixations. The CI images shifted along the y-axis since the number of fixations was different for each CI image computation. The data clearly showed that participants focus on 2 or 3 AOIs, and neglected the rest. Importantly, participants' selected AOIs differed to some degree. Three of the images used by PA were in the upper row, the only looked-at CT image in the bottom-row receiving 7 fixations only. Both PB and PC spent time looking at two CT images shown in the lower row; but the favorite CT image of PB was yet another CT image in the lower part. These results conclusively showed that the presentation of eight CT images was redundant for the participants.

Taking the above results into account, SI has been modified and the number of CT images displayed for entry and target point selection for kidney cryoablation was reduced to two.

### Evaluation of modified SI results

**Figure 7:**
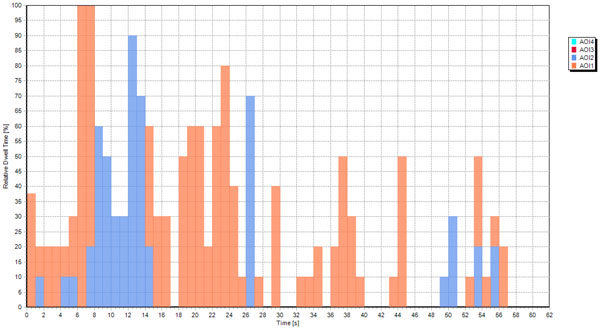
**Temporal evolution of dwell time for PD during interaction with the modified SI**.

We had prepared the modified SI with two scans - one axial and one coronal - only based on the results obtained with the early SI version, where we had presented eight axial scans simultaneously (Figure 3). The temporal distribution of dwell time for one participant (PD) is presented in Figure 7. Orange bars represented the relative dwell time with respect to the total task time on axial CT images on the SI, and the blue bars represented the relative dwell time with respect to the total task time on coronal CT images. Even though the results from this participant suggest that the presentation of two different types of scans is important, the other two subjects mostly looked at only the axial scan, which is discussed below.

When SI had been revised, time required to complete the task by the participants had been decreased (Table 1). In the first set of recordings, the three participants (PA, PB, and PC) had completed the task in 100, 72, and 159 seconds, respectively. When the SI had been modified, the participants (PD, PE, PF) needed only 62, 38 and 32 seconds, respectively, to complete the task. Even though these small sample sizes do not allow statistical testing, the results revealed that participants completed the task in less time with the new version of the SI.

**Table 1:** Total Time Each Participant Spent During Task Execution.

	Total Time	Relative time on CT images	Median Fixation Duration
**PA**	100s	**61 %**	168 ms
**PB**	72s	**55 %**	172.5 ms
**PC**	159s	**59 %**	169 ms
**PD**	62s	**28 %**	144 ms
**PE**	38s	**73 %**	262 ms
**PF**	32s	**79 %**	302 ms

**Figure 8:**
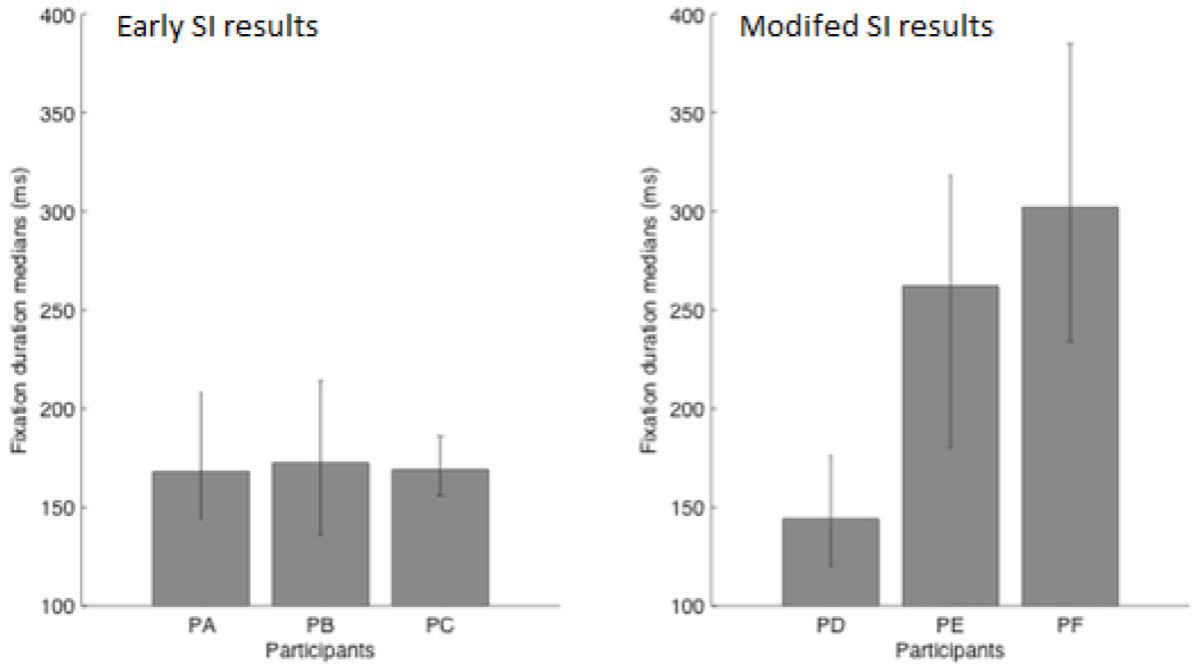
**Median Fixation Durations for Eary SI and Modified SI**.

The amount and duration of fixations on displayed CT images displayed had shown differences after the SI was modified. The participants had spent 61%, 55%, and 59% of their total viewing time fixating the CT images while interacting with early SI. The fixations of two participants covered 73% (PE) and 79% (PF) of viewing time, indicating a relative increase in visual information acquisition after SI had been modified. Surprisingly, this statistic was a modest 28% for the PD because PD had used the sliders for changing the zoom level and the contrast of CT images frequently. A closer inspection of the fixation properties revealed that whereas PD made frequent use of both axial and coronal CT images (63 and 28 fixations, respectively), the other two participants (PE and PF) rarely visited the coronal CT images (one and four fixations on the coronal image respectively). Moreover, for PE and PF, the fixation durations were relatively longer, which suggested, that they received more continuous information upon the initiation of a fixation. Permutation tests revealed that the fixation durations of PE and PF were longer than the durations observed for the participants during the early SI (*p*s < 0.05). The fixation durations of the PD, on the other hand, were not significantly different than the fixation durations measured with the early SI (all ps > 0.2). Figure 8 presented the fixation durations of all subjects together with 95% bias corrected bootstrap confidence intervals. Thus, the decrease in the number of CT images presented on the SI led to shorter task execution times, and for two of the three subjects (PE and PF), the fixation data suggested that they benefited only from the axial CT image with relatively long lasting fixations.

**Figure 9:**
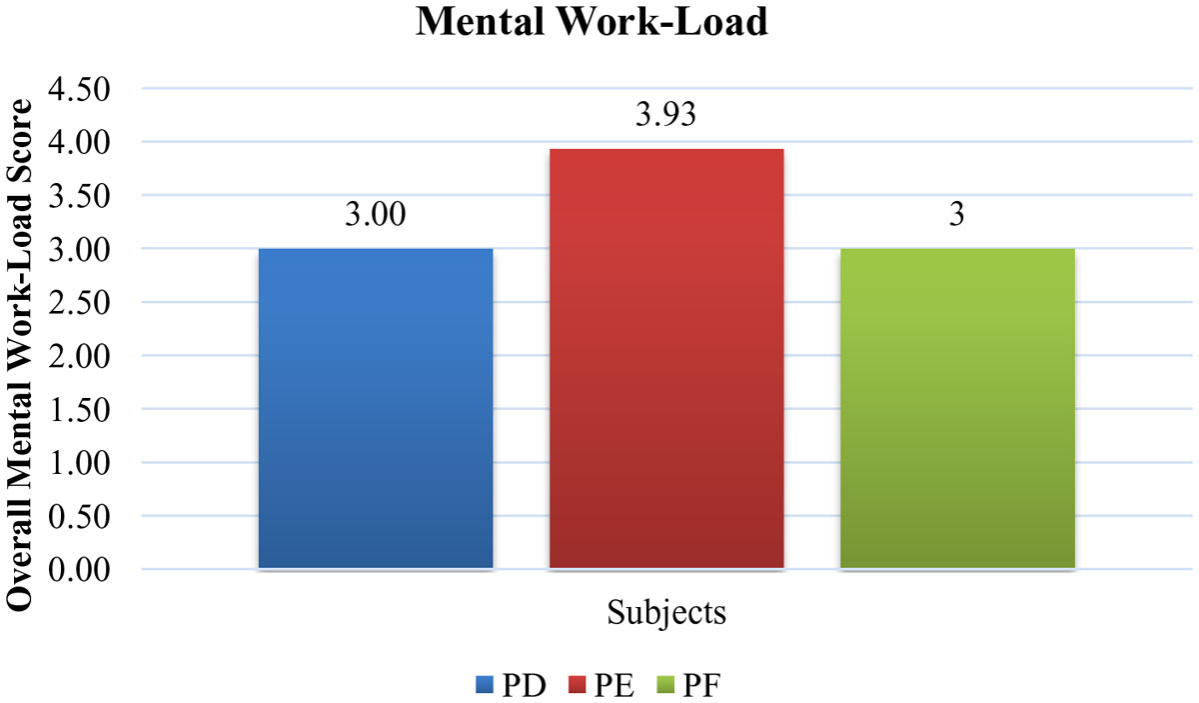
**Overall Mental Workload Scores for PD, PE and PF**.

**Figure 10:**
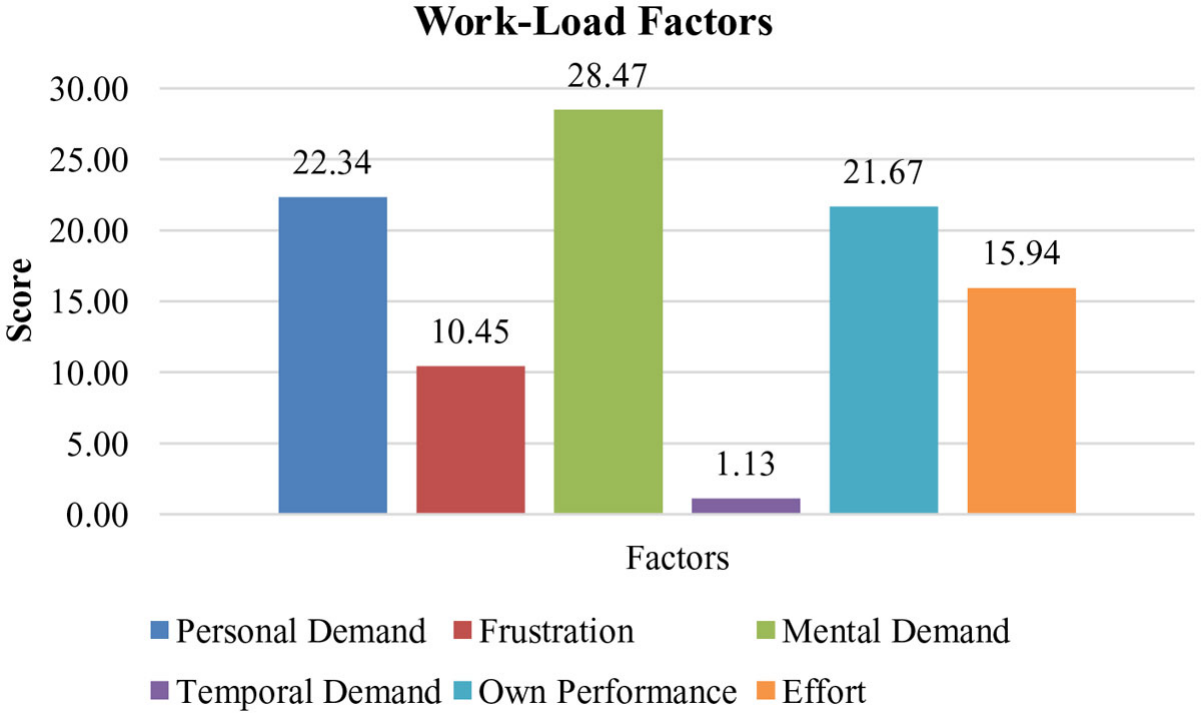
**Workload Factors Scores of Each Dimension**.

Overall mental workload and situation awareness of participants had been evaluated when recordings with the modified SI were completed. Overall mental workload scores of the PD, PE and PF had been presented in Figure 9. The workload factor scores of each dimension, which were mental demand (MD), physical demand (PD), time demand (TD), effort (EF), performance (OP), and frustration (FR), had been shown in Figure 10. The results showed that overall mental workload of surgeons related with SI is low as it was aimed. However, mental demand formed nearly 29% of overall workload among all other factors. In order to decrease workload more, there was need to ease the activities related with mental demand such as thinking, deciding, calculating, remembering etc. Therefore, some changes such as different screen layout designs, graphic/text mixes, color combinations, icons may be done in the design.

**Figure 11:**
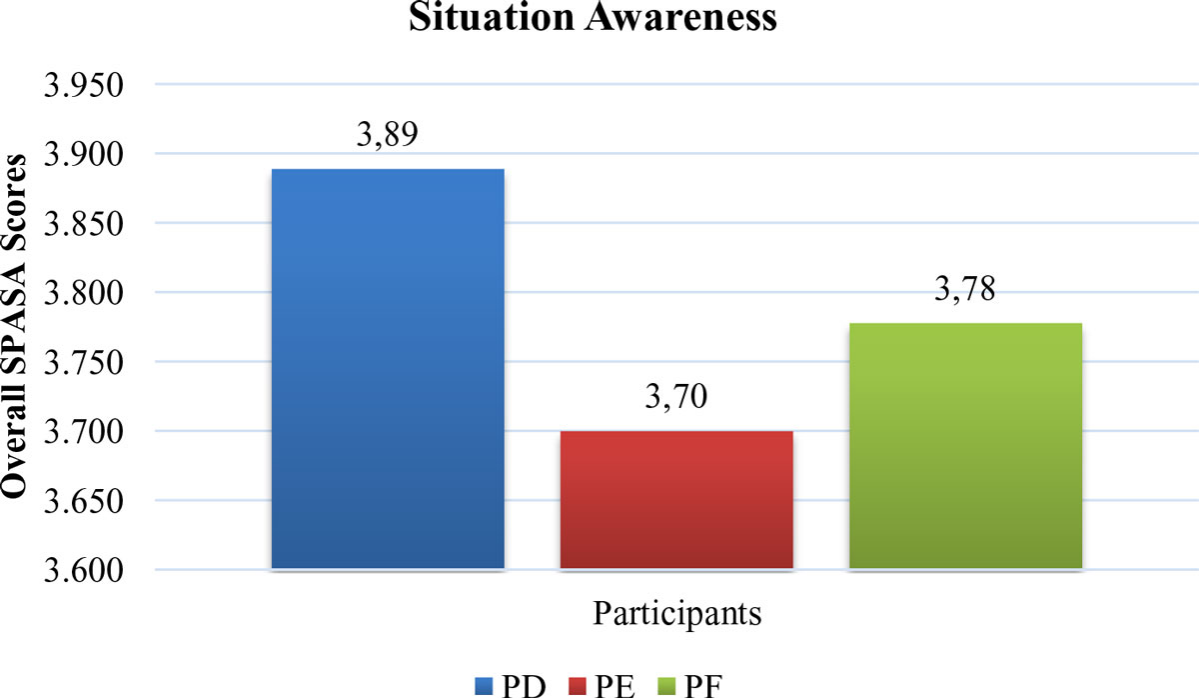
**Overall Situation Awareness Scores for Participants PD, PE and PF**.

**Figure 12:**
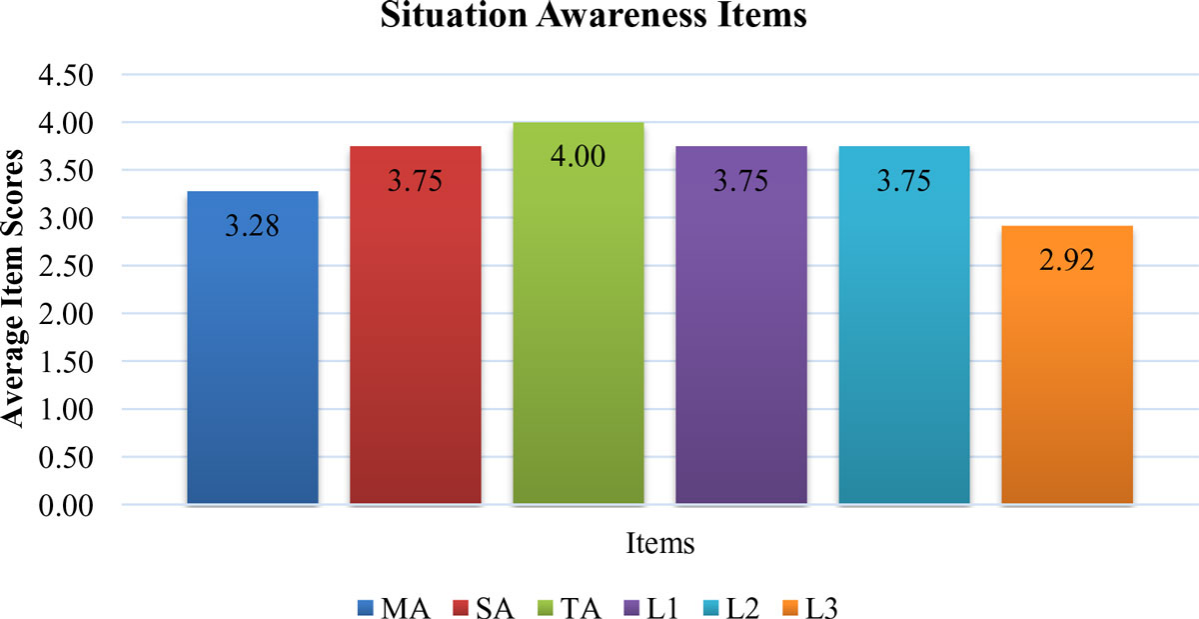
**Situation Awareness Items for Participants PD, PE and PF**.

Overall SA scores of surgeons were given in Figure 11. The results showed that SA of surgeons was considerably high. However, there was a little need to improve SA in terms of L3 (Figure 12). L3, which was the ability to predict actions in the future, was relatively low among all items. L3 was about how good the surgeons make predictions for future actions. Necessary knowledge and information about the process must be provided for surgeons to meet their objectives during the operation. They might have some questions related with SI and could not predict their future movements.

## Discussion

A surgical interface design may increase the effectiveness of a surgeon's performance during surgical operation. In this work, a surgical interface (SI) for kidney tumor cryoablation task, which considers the surgeon at the center, has been designed, developed and evaluated. A user-centered design (UCD) approach has been used to design the SI. The factors of usability and functionality have been considered during the design of SI. These factors have been selected and classified on the basis of a literature review and the personal judgement of the experts [24]. Possible configurations of the SI, which comprise various combinations of menu-based command controls, visual display of multi-modal medical images, 2D and 3D models of the surgical environment, graphical or tabulated information, visual alerts, etc., have been developed.

An eye tracking technology is used to improve the design of SI to obtain the optimum configuration. An eye tracking device, which provides quantitative data, is used to understand visual and display-based information processing, and the factors that may impact upon the usability of SI. Eye tracker devices provide a comprehensive approach while examining interaction processes like the ones in our study. Eye tracking studies had previously been used to highlight a surgeon's vigilance level during an operation [17], and to assess skill and situation awareness [33]. Additionally, it has been shown in [33] and [34] that workload assessment can be retrieved via an eye tracking experiment. Nevertheless, to the best of our knowledge, the current study is the first to use eye tracking data in order to introduce changes in a surgical interface, and below we discuss the significance of our findings in relation to previous studies.

We would like to mention one possible source of criticism that might be directed at present results before we discuss the significance of our findings,. In our study we had a small number of participants - three of them interacted with the early SI, and another three with the modified SI. Even though we accept that a larger participant sample is needed for more conclusive statistical analysis, we would like to note that in the case of eye-movement data, this sample is sufficiently informative. This is because our data points consist of fixations and not of subjects. Since each subject provides hundreds of fixations to the data pool, the analyses performed return reliable results. However, we intend to invite more participants for our future studies in order to see whether the results obtained from surgeons tested here generalize to others.

Analysis and interpretation of eye-tracking data is not straightforward [11]. The selection of both metrics that characterize gaze patterns and the statistical tools to investigate such metrics can be a daunting task. The gaze patterns of radiographers inspecting x-rays have been analyzed and revealed nearly 90.000 different associations between gaze patterns, stimulus statistics, task performance and the expertise of the user [14]. It is impossible to make sense of such an enormous data set without subjective manual filtering of the associations. In the present study, we have used only two simple fixation metrics - fixation duration and amount of fixations in task-relevant regions-with a clear goal in mind: The modification of our SI configuration such that surgeons interacting with it will solve a simulated cryoablation task faster and with more natural eye-movements [19]. We were able to conclude that the presence of eight scans in the early SI version was redundant by using the spatial distributions of fixations. This allowed us to present two larger scans at much higher spatial resolution, and consequently the task completion task decreased substantially. Moreover, as a result of this modification, fixation durations of the participants became similar to what is observed under natural viewing conditions. This latter point is especially interesting from a visual information acquisition perspective. Whereas long fixation durations might indicate difficulties in extracting information from an image region [35], many successive fixations of relatively short duration are associated with inefficient visual search [35]. We suggest that the durations recorded during interaction with the modified SI correspond to optimal levels. Thus, visual interface designs can be improved by considering natural eye movement properties.

The modified SI configuration also led to increases in total fixation time on CT scans that are crucial for determining the entry and target points during the simulated cryoablation task. Both studies on every day behaviors such as playing cricket [36] and research conducted in surgery training environments [37] demonstrate that expertise in a task correlates with increased fixation durations at task relevant regions of the environment. Even though we did not perform a comparison between novices and experts, the increase in fixation durations at CTs might suggest that the modified SI allowed our participants to channel their task-relevant expertise more effectively.

We have used NASA-TLX and SPASA questionnaires to measure surgeons' cognitive load and situation awareness, respectively during the execution of a kidney tumor cryoablation task with the help of a SI. The questionnaire results have shown that overall mental workload of surgeons related with SI is low as it has been aimed. However, mental demand has formed nearly 29% of overall workload among all other factors. In order to decrease workload more, some changes such as different screen layout designs, graphic/text mixes, color combinations, icons should be done in the SI design. Furthermore, overall SA scores of surgeons have been considerably high. However, there was a little need to improve SA in terms of L3. Thus, necessary knowledge and information about the process should be demonstrated to the surgeons by SI to meet surgeons' objectives during the task execution. In this study, it is possible that participants might have some questions related with SI, and could not predict their future movements.

## Conclusions

This preliminary study highlights the design of a SI using a user-centered approach (UCD) and evaluation of developed SI using subjective (questionnaires) and objective (eye tracking) methods to obtain the optimum SI configuration. The present results provide a proof-of-concept that user interface designs configured according to eye movement characteristics lead to improved usability.

## Future work

In the future, we plan to continue revision of the developed SI, include the real-time cryoablation protocol, and plan to collect eye tracking data in more difficult surgery-like tasks such as cryoneedle control. We believe varying surgery-like tasks will yield richer data on the eye tracking. We plan to add saccade properties and computational attention modeling to our eye tracking analysis [38], [39]. Interface designs considering eye movement characteristics might lead to improved user comfort by avoiding saccade directions and amplitudes uncommon in everyday behaviour. Computational attention models can predict fixation locations of human subjects by relying on bottom-up saliency computed from low-level visual properties such as contrast and color [40]. The saliency of informative regions may be increased to transmit the relevant information to the surgeon as quickly as possible based on this computational attention models. Moreover, it will be interesting to characterize the contributions of stimulus-driven saliency and the top-down expertise of surgeons to their gaze behavior [41]. Thus, the investigation of the eye movements of surgeons interacting with our SI will be important both from usability and basic attention research perspectives. Furthermore, one can compare the fixations of the surgeons performing the task with fixation locations generated by computational models of visual attention (e.g. [40]) to improve the visual design of the developed SI.

We also plan to investigate the use of eye tracking system to measure surgeons' mental workload and situation awareness during kidney tumor cryoablation task execution with the SI. Eye tracking observations such as pupil size, duration of fixation, saccade amplitude can be used to assess mental workload and SA. Eye tracking and questionnaire results (NASA-TLX and SPASA) can both be used to obtain the optimum SI configuration to decrease mental workload and to increase situation awareness of the surgeon.

## Competing interests

The authors declare that they have no competing interests.

## Authors' contributions

DEB conceived and designed the research. DEB and AA performed the research including data collection, testing and analysis. DEB suggested extensions and modifications to the research. DEB supervised the whole research, and revised the manuscript critically. All authors read and approved the final manuscript.

## Declarations

Publication of this article has been funded by European Union Seventh Framework Programme FP7/2007-2013 under grant agreement n. 270396 (I-SUR).

This article has been published as part of *Theoretical Biology and Medical Modelling *Volume 11 Supplement 1, 2014: Selected articles from the 1st International Work-Conference on Bioinformatics and Biomedical Engineering-IWBBIO 2013. The full contents of the supplement are available online at http://www.tbiomed.com/supplements/11/S1.
