# Erratum to: improvement of design of a surgical interface using an eye tracking device

**DOI:** 10.1186/1742-4682-11-48

**Published:** 2014-11-03

**Authors:** Duygun Erol Barkana, Alper Açık, Dilek Goksel Duru, Adil Deniz Duru

**Affiliations:** Electrical and Electronics Engineering Department, Yeditepe University, Istanbul, 34755 Turkey; Psychology Department, Özyeğin University, Istanbul, 34794 Turkey; Biomedical Engineering Department, Istanbul Arel University, Istanbul, Turkey; School of Physical Education and Sports, Marmara University, Istanbul, Turkey

## Correction

After publication of this work [1], we noted that we inadvertently failed to include the complete list of all co-authors. The full list of authors has now been added and the Authors’ Contributions and Competing Interests sections modified accordingly.
